# Wireless Motion Sensors—Useful in Assessing the Effectiveness of Physiotherapeutic Methods Used in Patients with Knee Osteoarthritis—Preliminary Report

**DOI:** 10.3390/s20082268

**Published:** 2020-04-16

**Authors:** Jagoda Goślińska, Agnieszka Wareńczak, Margaret Miedzyblocki, Krystyna Hejdysz, Ewa Adamczyk, Paweł Sip, Ewa Chlebuś, Jarosław Gośliński, Piotr Owczarek, Adam Woźniak, Przemysław Lisiński

**Affiliations:** 1 Department of Rehabilitation and Physiotherapy, University of Medical Sciences, 28 Czerwca 1956 Str., No 135/147, 60-545 Poznań, Poland; agnieszka.warenczak@gmail.com (A.W.); miedzyblocka.m@gmail.com (M.M.); krystynahejdysz@gmail.com (K.H.); adamczykewaagnieszka@gmail.com (E.A.); pawel.sip@gmail.com (P.S.); ewchl@gazeta.pl (E.C.); plisinski@vp.pl (P.L.); 2 Aisens Sp. z o. o., Lubeckiego 23A, 60-348 Poznań, Poland; j.goslinski@aisens.co (J.G.); p.owczarek@aisens.co (P.O.); a.wozniak@aisens.co (A.W.)

**Keywords:** wireless sensors, mobile applications, proprioception, knee joint, osteoarthritis

## Abstract

Osteoarthritis of the knee (OAK) is characterized by pain, limitation of joint mobility, and significant deterioration of proprioception resulting in functional decline. This study assessed proprioception in OAK patients following two ten-day rehabilitation programs using the Orthyo^®^ system. Fifty-four study participants with clinical symptoms and radiological signs of OAK were randomly divided into an exercise group (*n* = 27) or a manual therapy group (*n* = 27). The control group consisted of 27 volunteers with radiological signs of OAK, but with no clinical symptoms or prior history of rehabilitation. The following parameters were assessed: knee proprioception using inertial sensors and a mobile application, patients’ function using Western Ontario and McMaster Universities osteoarthritis index (WOMAC), and pain intensity using the visual analog scale (VAS). Following rehabilitation, knee proprioception tests did not improve in either study group. Both study groups showed significant improvement of the WOMAC-assessed function (exercise group: *p* < 0.01, manual therapy group: *p* = 0.01) and a significant decrease (*p* < 0.01) of VAS-assessed pain following rehabilitation, but the post-therapy results did not differ significantly between the aforementioned groups. The Orthyo^®^ system provided a quick and accurate assessment of the knee joint position sense. There was no direct relationship between functionality, pain, and proprioception threshold in the knee joint.

## 1. Introduction

Osteoarthritis of the knee (OAK), even with unilateral presentation of symptoms, causes a significant deterioration of function, which, in turn, lowers patients’ quality of life. In broader terms, it poses a significant public health problem [1,2,3].

As reported by Mahmoudian et al. [4], OAK is highly prevalent in people above the age of 60, and it is typically associated with pain, stiffness, muscle weakness, and proprioceptive deficits. These proprioceptive deficits increase the risk of falls and other injuries in OAK patients, leading to disability and worsening of quality of life [5,6]. Some authors claim that proprioceptive deficits trigger OAK [7,8]. These findings stand in contrast to a study by Baert et al. [9], which shows that changes in kinesthesia result from OAK rather than cause it, and therefore, proprioceptive exercise does not inhibit the development of OAK. Findings by Baert et al. are supported by van der Esch et al. [10], who report that OAK causes proprioceptive deterioration, which increases pain and reduces patient activity level. Furthermore, Chen et al. [11] observed that in OAK cases, increased pain, lower limb muscle weakness, and functional deterioration were associated with knee proprioceptive deficit.

Proprioception can be evaluated by various methods, and for the knee, a joint position sense (JPS) test is commonly used [7,8,12,13]. The JPS test involves a position matching procedure in which a target joint position is presented, and the test subject must actively recreate that position (so-called end angle) [14]. JPS is usually measured by goniometers, digital photography, or a camera-based system [15,16,17,18]. We found only one study that used inertial sensors to test JPS [13]. This method is accurate, repetitive, and easy to administer and thus appears to have an advantage over the commonly used methods.

A review by Knoop et al. [7] revealed that JPS disorders represented a significant risk factor for dysfunction progression in OAK patients, thus contributing to poorer quality of life, while rehabilitation treatment (exercise) might slow down the dynamics of this process. Therefore, Sanchez-Ramirez et al. [19] proposed that JPS examination and implementation of appropriate exercises in cases of proprioceptive deficits should become a permanent feature of the rehabilitation process in OAK patients. Furthermore, Collins et al. [20] proved that improving the knee JPS could slow down the progression of osteoarthritis by optimizing load distribution acting on the articular cartilage, which correlated with better functional outcomes reflected in Western Ontario and McMaster Universities osteoarthritis index (WOMAC). Therefore, preferred treatment methods for the aforementioned disorder are those that reduce excessive load on the knee joints, thus reducing pain intensity while maintaining daily activity at an optimal level.

Among various rehabilitation methods, proprioceptive exercises that improve knee joint stability and functionality deserve a special recommendation [21]. However, to date, there has been limited research focused on analyzing the effectiveness of kinesitherapy on joint position sense in OAK patients [22,23,24,25]. Bearing in mind the distribution of deep sensation receptors in various knee structures [26], theoretically, it can be assumed that almost every kinesitherapy technique should exert some positive influence in this respect [27]. However, a question arises whether the different kinesitherapy techniques are equally quick and efficient in improving JPS. Most researchers agree that the earliest positive effects of a specific exercise of OAK may be observed following a four-week exercise regimen [28,29]. Still, it is not known whether other therapy techniques, which do not necessarily require active patient participation and last less than four weeks in duration, could also have a significantly positive impact on knee JPS. This matter is crucial because, in the clinical practice, the time required to achieve the desired therapeutic outcome determines the overall therapeutic success, understood as a global function of the patient [30].

Therefore, in this study, we aimed to assess the usefulness of wireless motion sensors coupled with a mobile application in evaluating the knee JPS. We also evaluated the degree of knee joint deviation from its correct trajectory during flexion, which we did not find to have been analyzed in the literature so far. We used these parameters to compare the effectiveness of two therapeutic methods—manual therapy and exercise. The former method focused on the treatment administered to patients by a physical therapist, which included soft tissue and joint techniques. The latter method relied on patients actively partaking in an exercise program aimed at improving proprioception. We evaluated which of the two ten-day rehabilitation programs used in OAK patients was more effective in improving the knee JPS and in supporting the correct execution of knee flexion along its physiological trajectory. In addition, we investigated whether the two implemented rehabilitation programs affected pain intensity and the overall function of patients with OAK.

## 2. Materials and Methods

### 2.1. Participants

A randomized controlled trial of patients with OAK was carried out between December 2018 and April 2019 at the Outpatient Rehabilitation Ward of Wiktor Dega Orthopedic-Rehabilitation Clinical Hospital, Poznań University of Medical Sciences. The study group consisted of 54 patients with OAK (grade I and II, according to the Kellgren-Lawrence classification system) [31]. Patients reported at least three months of foregoing, exertional knee joint pain, and limitation of function during their baseline physical examination upon admission to the Outpatient Rehabilitation Ward. Initially, we included 116 patients, but due to the exclusion criteria, we had to disqualify 35 of them. 

Patients with the following conditions were excluded from the study:

1. advanced osteoarthritis of the knee (grade III or IV according to the Kellgren-Lawrence classification system),
2. neurological diseases,
3. rheumatic diseases (rheumatoid arthritis, ankylosing spondylitis, psoriatic arthritis),
4. sciatica,
5. assisted gait,
6. any congenital or acquired lower limb deformity,
7. prior surgery that could affect knee joint function (anterior cruciate ligament reconstruction, meniscectomy, total knee replacement, total hip replacement, osteotomy, and arthrodesis of the lower limb).

Study participants were randomly divided into two groups (*n* = 27) and treated either with synergistic exercises in a closed kinematic chain (E) or with manual therapy (MT). The control group (C) consisted of 27 volunteers, matched for sex, age, and radiological signs of OAK, but with no clinical symptoms or prior history of rehabilitation. All participants were evaluated prior to study commencement and at its conclusion ten days later. Written consent of participation in the study was obtained from each participant following a detailed explanation of study aims and methodology. Characteristics of basal anthropometric data of the study population are shown in Table 1. Kruskal-Wallis test was used to analyze the differences between the three groups. No significant differences in age (*p* = 0.10), height (*p* = 0.52), weight (*p* = 0.69), or Body Mass Index (BMI) (*p* = 0.07) were found between the groups.

The study was conducted in accordance with the Declaration of Helsinki and with the approval of the Ethics Committee of the Karol Marcinkowski University of Medical Sciences in Poznań (No. 522/18).

### 2.2. Sensors and Application

The Orthyo^®^ system (Aisens sp. z o.o. Poznan, Poland) is a certified class Im (I measure) device according to the medical device classification system used in Poland. It uses three basic types of sensory data: acceleration, velocity, and magnetic field. Raw sensory data collected by the sensor are filtered, calibrated, and computed in an estimation process by the sensor’s microchip. As a result, the sensor generates quaternions—orientation and relative position. The sensors’ estimate is given in a referential system whose axes are positioned following the East North Up (ENU) principle, where X points eastwards, Y northwards, and Z upwards. Estimation and calibration are based on such estimators as Kalman filter, complementary filters, and supporting artificial intelligence algorithms. All computed data are sent to the Orthyo-App via Bluetooth low energy, initiating the second stage of data processing. During this stage, all interrelationships between sensors are computed, yielding parameters, which represent the movement of a specific joint (e.g., data from sensors located on the thigh and calf are used to determine knee joint parameters). The second step of data processing computes linear velocity, acceleration, movement in space, and enables frequency analysis, providing information about, for example, changes in knee position in the three planes. The archiving and retrieving system provides access to cloud-stored data, enabling web-based tracking of results. The detailed specification of the Orthyo^®^ system is described in the previous article [32].

### 2.3. Experimental Procedures and Instruments

Prior to the therapeutic intervention, all patients were evaluated using three outcome measures: proprioception tests executed with the Orthyo^®^ system under open kinematic chain conditions (the end segment of the limb was free), self-administered health status measure—the WOMAC measure, and the visual analog scale (VAS).

#### 2.3.1. Proprioception Tests (Orthyo^®^ system)

**Figure 1:**
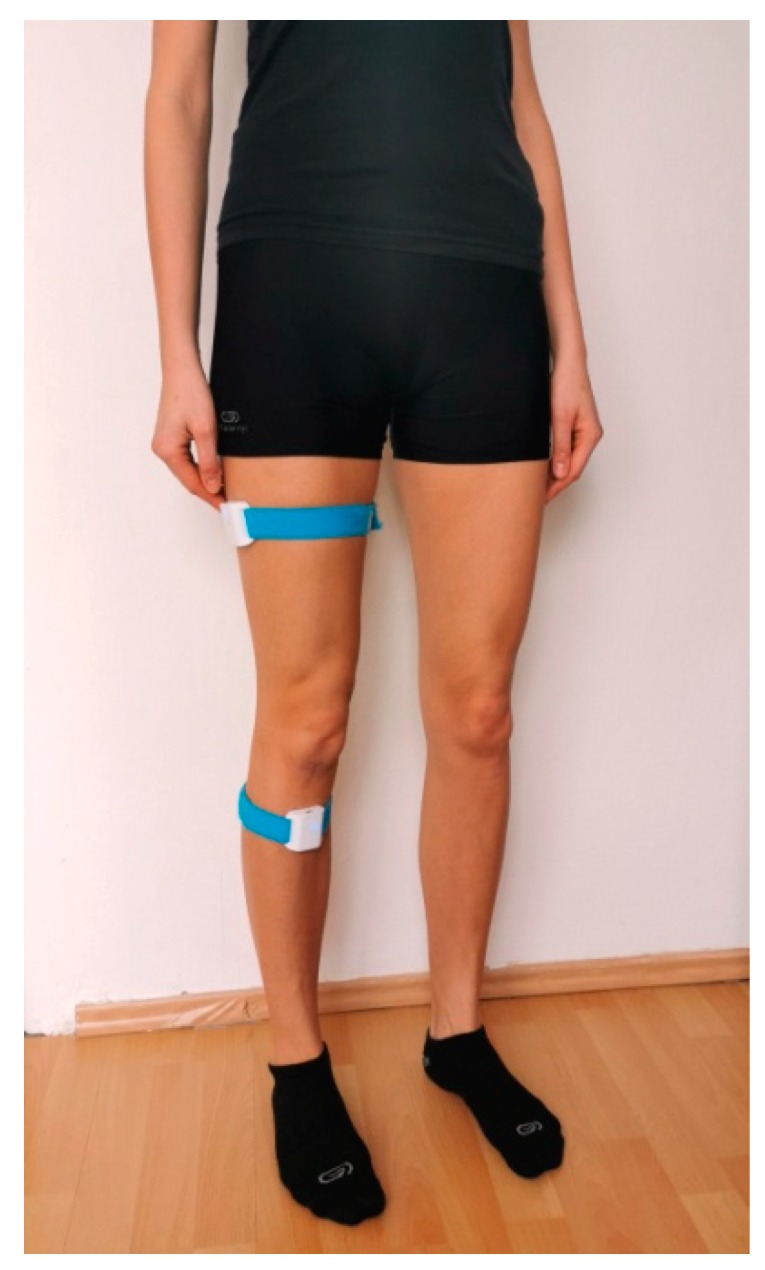
Placement of Orthyo^®^ system sensors on the lower extremity.

Prior to commencing the proprioception tests, each patient was registered in the web panel. Next, four Orthyo^®^ system sensors were placed on both patient’s lower limbs and secured with velcro bands. One sensor was attached to the lateral surface of the thigh, 15 cm distally to the greater trochanter, while the other sensor was placed on the anterior surface of the shin, 5 cm distally to the tibial tuberosity. The model for attaching sensors on the limb is shown in Figure 1. The sensors were used in conjunction with a mobile application installed on a smartphone fitted with the Android 5.0 lollipop operating system. Upon commencing the proprioception tests, this application was used to pair the smartphone with the sensors using Bluetooth technology.

#### 2.3.2. WOMAC

WOMAC is a self-administered health status measure originally developed for use amongst patients with knee and/or hip osteoarthritis. The WOMAC survey is comprised of 24 items divided into three subscales: pain (5 items), stiffness (2 items), and physical function (17 items). Each test question is scored on a scale of 0–4, and the values are summed up for a combined maximal score of 96. Higher scores on the WOMAC indicate worse pain, stiffness, and functional limitations. The WOMAC survey has been demonstrated to be valid, reliable, and responsive and has been used extensively both in research studies and clinical trials [33].

#### 2.3.3. VAS

The visual analog scale is a unidimensional measurement instrument that gauges pain intensity across a continuum of values. It is presented as a 100-mm horizontal line on which the patient’s pain intensity is represented by a point between 0 (no pain at all) and 10 (worst pain imaginable). A higher VAS score indicates greater pain intensity. Its simplicity, reliability, and validity make VAS the optimal tool for describing pain severity or intensity [34].

### 2.4. Testing and Intervention Procedures

#### 2.4.1. Testing

Proprioception was measured by two parameters prior to and at the conclusion of a ten-day rehabilitation program. The first parameter—joint position sense evaluated test subjects’ ability to recreate, without visual modality, a target knee joint position presented by the examiner. With the test subject in a prone position, the examiner passively flexed the subject’s knee joint to 60 degrees. The subject held this position for five seconds and then fully extended the knee. The subject was then asked to actively recreate the previously presented target joint position and hold it for a duration of two seconds. The application recorded the recreated angle (end angle) and then calculated the difference between it and the target angle. This open kinematic chain test was performed once for each knee joint.

The second parameter—the mean square error (*MSE*) is the mean squared error from the knee joint trajectory in the sagittal plane expressed in ${\left({}^{\circ}\right)}^{2}$ and calculated using the following formula:

$$MSE=\frac{1}{n}\overset{n}{\underset{i=1}{\sum }}r_{i}^{2}$$

where “*r*” is the deviation angle from the initial sagittal plane to the actual sagittal plane. 

#### 2.4.2. Intervention

**Figure 2:**
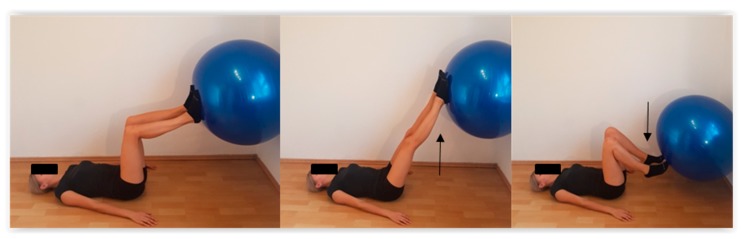
Exercise protocol for the ball-rolling task.

The E group study subjects were treated with a closed kinematic chain synergistic exercise regimen, which involved rolling a ball against a wall using feet while lying supine, with the feet resting firmly on a ball. The study subjects were asked to keep their hips flexed at 90 degrees but were free to flex and extend their knees while performing the rolling exercises (Figure 2). 

**Figure 3:**
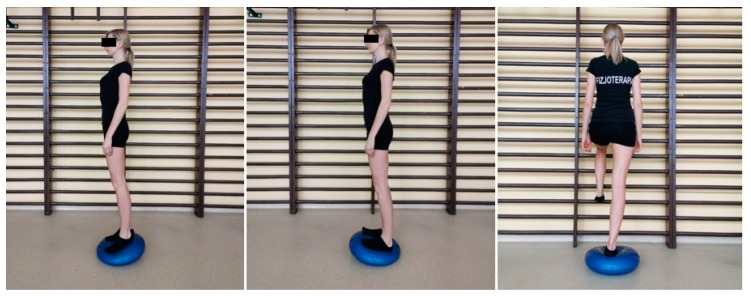
Exercises on the sensorimotor cushion.

Additionally, E group test subjects performed closed kinematic chain balance exercises while standing upright on sensorimotor cushions (Figure 3). The MT group study subjects were treated with manual therapy, which involved patella mobilization and deep tissue massage. The group-specific interventions were repeated daily for ten consecutive days. The therapy was performed by five qualified physiotherapists, who followed the same procedures based on previous arrangements. Control group subjects had no therapy of the knee joint.

### 2.5. Statistical Analysis

Data were analyzed with Statistica™ software version 13.1. Descriptive statistics were reported as mean, standard deviation (SD), median, and range. The Shapiro–Wilk test was used to assess the normality of distributions in the test scores. The dependent t-student test or Wilcoxon’s signed-ranks test was conducted to compare the differences between results obtained before and after treatment. Nonparametric Mann–Whitney U test was used to analyze differences between the two groups. Kruskal-Wallis test was used to analyze the differences between the three groups. A posthoc analysis was used in the cases when there were statistically significant differences in the measures. The *p*-values of less than 0.05 were considered statistically significant and highlighted in tables using the red color.

## 3. Results

### 3.1. Proprioception Measurements

Pre- and post-study proprioception tests’ results for both therapeutic intervention groups and the no-intervention control group were compared. The JPS and MSE results obtained for both the right and the left knee joint did not differ significantly between the three groups prior to study commencement. However, following the study conclusion, the left knee proprioception differed significantly between the groups (*p* = 0.04). Posthoc analysis revealed this difference to be present between the two therapeutic intervention groups. In the MT group, the end angle was, on average, 5.3 degrees further away from the target angle of 60 degrees than in the E group.

Moreover, the intragroup left knee JPS for the MT subjects differed significantly pre- and post-study (*p* = 0.02). Following treatment, MT group subjects achieved significantly poorer JPS results than pre-treatment, averaging 72.7 and 67.0 degrees, respectively. No statistically significant results were obtained for the right knee JPS or MSE in either intergroup or intragroup comparisons. The data are presented in Table 2.

### 3.2. WOMAC

Pre- and post-study WOMAC scores for both therapeutic intervention groups and the no-intervention control group were compared (Table 3). Prior to study commencement, the control group obtained significantly lower (better) (*p* < 0.01) scores than either of the study groups. However, the MT and E groups did not differ significantly from each other. Following the study conclusion, WOMAC scores in both study groups dropped significantly (E: *p* < 0.01, MT: *p* = 0.01), which translated into a significant functional improvement within each study group, but there was no significant difference in post-study WOMAC scores between the E and MT groups.

### 3.3. VAS

Pre- and post-study VAS scores for both therapeutic intervention groups were compared (Table 4). Prior to study commencement, the MT and E groups did not differ significantly from each other. Following the study conclusion, VAS scores in both study groups dropped significantly (*p* < 0.01), which translated into significant pain reduction within each study group, but there was no significant difference in post-study VAS scores between the E and MT groups.

## 4. Discussion

Our study assessed the effectiveness of two physiotherapeutic techniques on the proprioception parameters in OAK patients. Proprioception is one of many features that should be considered in a complex evaluation of the patient with the OAK. We used inertial sensors, which, in the past, were rarely used for proprioception testing.

Goniometers, both electric [24,35,36] and mechanical [37,38], are commonly used tools for assessing joint position sense. However, goniometer results in users’ risk measurement errors due to incorrect fulcrum positioning. Additionally, simultaneous alignment of the goniometer’s two arms with the bones forming the joint may hinder free joint movement, thus affecting measurement accuracy [39]. Some researchers combine a goniometer examination with photography, which certainly increases the measurement accuracy, but is very time-consuming [15]. Other types of research have evaluated JPS using camera-based motion capture systems. These systems are highly accurate but pricy and requiring specific conditions and sophisticated measuring equipment [16,18]. In our study, we determined JPS under open kinematic chain conditions using two inertial wireless sensors in conjunction with a mobile application, which permitted unhindered joint movement. Additionally, proprioception can be assessed through one’s ability to maintain knee joint stability in the frontal plane [32]. We measured this frontal plane stability under closed kinematic chain conditions by assessing the degree of knee joint deviation from its correct trajectory in the frontal plane during flexion. We expressed it via the MSE parameter [32]. The literature search yielded no studies citing the use of this parameter in relation to specific disease entities. We employed the described methods in clinical practice because OAK patients develop a number of undesirable anatomical changes that negatively affect the knee joint position sense [27,40,41] and cause pain [21,26] and functional deficits [2,3,7,12,30]. Therefore, based on available data from other studies [27,42], we determined that the treatment algorithm for OAK patients should include therapeutic procedures that improve joint position sense and overall function while reducing pain intensity.

One such procedure, often used to manage OAK patients, is a closed kinematic chain synergistic exercise regimen for the lower limb [27,35,41]. This therapeutic procedure is characterized by direct contact of the lower limbs with the ground or exercise equipment during the therapeutic session. Exercises of this type also increase knee joint stability [43,44,45]. Cho et al. attributed this effect to building dynamic stability through simultaneous contraction of antagonistic muscles, while concomitant mechanoreceptor stimulation led to an improvement in proprioception [44]. The effect of an absence of active stimulation in muscles producing knee joint movement on JPS deterioration has also been proven in other studies [35,42,46]. Jan et al. [47] noted that the advantage of closed kinematic chain exercise over open kinematic chain exercise in OAK patients was based on the fact that only the former improves JPS. This view was contradicted by Kachanathu et al. [44], who reported that open kinematic chain exercise also improved JPS in OAK patients, however, to a lesser extent than the closed chain counterpart [42]. Therefore, the closed kinematic chain exercise regimen we used in this study included active patient participation aimed at antagonistic muscle activation, which was consistent with the dominant view of closed kinematic chain use expressed in other publications [36,37,43,45]. Exercise equipment used during our exercise regimen included a Swiss ball and sensorimotor cushions. Similar exercise technique, with beneficial health outcomes for patients, was employed by Salaheldin and Hassanien [48] and Kumar et al. [36]. In turn, AdemolaGbiri et al. [42] and Lin et al. [35] used a different closed kinematic chain exercise technique in their studies, proving that alternative therapeutic approaches could positively affect the JPS in OAK patients as long as they are executed under closed kinematic chain conditions.

An interesting assessment of exercise effect in terms of its potential impact on knee JPS was presented by Younis et al. [49], who compared two kinesitherapy techniques: dynamic stretching and proprioceptive neuromuscular facilitation. Both techniques improved body balance but did not significantly affect the knee JPS. Among other therapeutic methods aimed at improving the knee JPS, there may be found programs using virtual reality [38], exercises using biofeedback [35], and exercises using the sling suspension system [50]. Conversely to other studies, which have shown results of the knee JPS improvement as a consequence of using a closed kinematic chain exercise program, we did not obtain similar results in our study (see Table 2). This might be due to the fact that our exercise program was only ten days long, while exercise regimens executed by other authors lasted at least four weeks [36] and, in most cases, eight weeks [35,37,47]. Based on the few available literature reports [51,52,53] that have emphasized on the appropriateness of using manual therapy in OAK, we used manual therapy in one of two study groups, with the assumption that improvement of joint range of motion and relaxation of perigenual muscles, ligaments, and fascia can improve the JPS. A literature search revealed that only Ko et al. [52] assessed the effect of manual therapy combined with active lower limb exercise on the JPS in OAK patients. The authors reported that a significant improvement occurred following eight weeks of such therapy. Our study results (see Table 2) did not show improvement of the knee JPS in the manual therapy study subjects following the conclusion of the ten-day rehabilitation program. This lack of JPS improvement might have resulted from a relatively short duration of therapy and from the fact that the perigenual soft tissue mobilization is not accompanied by active lower limb exercise.

The only study that supports the use of manual therapy without any other procedures in the treatment of OAK patients with regard to improving the knee JPS was presented by Yang et al. [26]. According to those authors, during knee mobilization performed at high speed and low amplitude, there is an intensive stimulation of mechanoreceptors located in the muscles, tendons, and the joint capsule, which results in JPS improvement. However, it must be noted that Yang et al. studied patients with cervical spine pain.

In both our study groups, we also assessed the impact of exercise and manual knee joint mobilization on the patients’ function and pain intensity, using the WOMAC scale and the VAS scale, respectively. Research by Sekir et al. [37], AdemolaGbiri et al. [42], and Tsauo et al. [50] showed that active lower limb exercise improved OAK patient function and reduced pain intensity regardless of the exercise technique. The results obtained in our study (see Table 3 and Table 4) were consistent with the observations of the aforementioned authors. Lower limb activating exercise performed under closed kinematic chain conditions had indeed improved patients’ function and reduced pain intensity following only a short, ten-day therapeutic program, which was a new finding because up till now, only longer periods of exercise have been evaluated in the literature [35,36,37]. However, we did not achieve an improvement of JPS in either of our therapeutic-intervention groups. Therefore, it can be assumed that pain and function do not directly impact the knee JPS, which was supported by Jan et al. [47], who led to a similar conclusion based on their research findings.

Although all assessed parameters are relevant, a truly comprehensive assessment of OAK patients would require an evaluation of other biomechanical parameters. Other authors have focused on muscle activation patterns and strength measured by EMG [54,55,56] and dynamometers [57,58], as well as gait analysis, measured via visual or inertial motion capture systems [59,60,61,62]. 

## 5. Conclusions

1. The Orthyo^®^ system used in conjunction with a mobile device fitted with the Android 5.0 Lollipop operating system allowed for a quick and accurate assessment of the knee joint position sense, which made it a great verification tool for evaluating the effectiveness of selected rehabilitation programs.
2. A 10-day rehabilitation regimen did not significantly affect the knee joint position sense, regardless of the type of therapeutic intervention used.
3. Even a relatively short (10-day) period of rehabilitation produced significant functional improvement and pain reduction, regardless of the type of therapeutic intervention used.
4. For a complete functional evaluation of the patient with OAK, muscle activity measurements and gait analysis should be added.

### Limitations

A relatively small number of study subjects may be considered a limitation of this study, but since this is the first study that assesses the clinical usefulness of the inertial sensor system presented, it may be considered pilot in nature. Moreover, the subjective nature of WOMAC-assessed function and VAS-assessed pain may cause concern. Those scales are largely self-assessment tools that may affect the reliability of the results obtained. Furthermore, the rehabilitation protocol used in our study may be considered too short in duration, when it is compared to studies presented by other authors. However, we believe that verifying the effectiveness of selected therapeutic interventions as early as possible is beneficial to our patients. Finally, inquiries may be made regarding our reasons for choosing to limit the therapeutic interventions to two types only. This protocol was chosen because we aimed to confirm the effectiveness of exercise under closed kinematic chain conditions in a shorter time frame than was presented in other studies and to supplement knowledge on the effectiveness of manual therapy targeting the knee joint since this efficacy has not been widely studied.

## Figures and Tables

**Table 1:** Main anthropometric data of the study population (only included patients).

Variable	Group	No.	Mean ± SD	Median	Min–Max	*p*-Value
age (years)	E	27	65.0 ± 7.4	64.0	55.0–78.0	0.10
	MT	27	66.1 ± 4.7	65.0	58.0–76.0	
	C	27	63.0 ± 6.6	60.0	53.0–76.0	
weight (kg)	E	27	76.1 ± 11.3	75.0	58.0–100.0	0.52
	MT	27	76.2 ± 11.5	75.0	58.0–105.0	
	C	27	73.5 ± 12.3	68.0	56.0–90.0	
height (m)	E	27	1.67 ± 0.07	1.66	1.53–1.82	0.69
	MT	27	1.66 ± 0.07	1.66	1.55–1.82	
	C	27	1.68 ± 0.07	1.67	1.56–1.80	
BMI (kg/m^2^)	E	27	27.3 ± 3.8	26.8	20.4–36.1	0.07
	MT	27	27.6 ± 4.0	26.9	20.9–38.3	
	C	27	25.9 ± 3.8	24.5	20.5–34.3	

*p*-value: the comparison of intergroup age, weight, height, and BMI (Kruskal-Wallis test) E – group treated with synergistic exercises in a closed kinematic chain, MT – group treated with manual therapy, C – control group

**Table 2:** Results of proprioception evaluation (end angle for JPS test and MSE) in E, MT, and C groups of participants.

Variable	Group	Before				After				*p*-Value^3^
		Mean ± SD	Median	Range	*p*-Value^1^	Mean ± SD	Median	Range	*p*-Value^2^	
End angle (°) Left	E	67.0 ± 9.3	66.6	51.6–87.0	0.31	67.4 ± 7.5 ^a^	68.1	52.9–86.4	0.04	0.84
	MT.	67.0 ± 9.1	66.7	46.3–85.8		72.7 ± 9.5 ^a^	71.4	48.2–101.7		0.02 *
	C	69.9 ± 6.5	72.3	54.9–77.7		69.5 ± 10.7	669.0	50.9–98.0		0.65 *
End angle (°) Right	E	68.4 ± 9.3	67.7	46.6–85.6	0.99	68.3 ± 8.4	69.1	44.2–86.7	0.88	0.63 *
	MT.	70.4 ± 12.9	68.4	53.0–107.6		68.8 ± 11.2	66.6	47.6–98.5		0.53
	C	69.1 ± 8.1	68.4	59.1–87.8		67.4 ± 7.0	70.5	51.5–76.6		0.29
$\mathrm{MSE}{\left({}^{\circ}\right)}^{2}$ Left	E	11.0 ± 13.6	4.0	0–40.8	0.38	6.8 ± 5.4	6.7	0–17.0	0.96	0.33 *
	MT.	8.4 ± 15.1	4.1	0.4–79.4		10.3 ± 16.5	3.6	0.3–78.2		0.27 *
	C	6.0 ± 8.7	3.1	0.1–32.3		9.7 ± 11.0	5.0	0.1–32.2		0.26 *
$\mathrm{MSE}{\left({}^{\circ}\right)}^{2}$ Right	E	6.6 ± 8.7	2.9	0.1–33.9	0.41	5.1 ± 7.1	3.3	0.1–36.4	0.47	0.39 *
	MT.	5.2 ± 5.5	4.0	0–27.1		5.2 ± 4.9	3.8	0.1–15.9		0.77 *
	C	9.6 ± 9.7	7.5	0.1–32.8		7.3 ± 7.0	6.2	5.90–25.8		0.22

*p*-value^1^—the comparison of intergroup end-angle and MSE pre-treatment (Kruskal-Wallis test); *p*-value^2^—the comparison of intergroup end-angle and MSE post-treatment (Kruskal-Wallis test); *p*-value^3^—the comparison of intragroup end-angle and MSE pre- and post-treatment (dependent t-student test or * Wilcoxon signed ranked test); ^a^—posthoc analysis.

**Table 3:** Results of WOMAC measure in E, MT, and C groups of participants.

Variable	Group	Before				After				*p*-Value^3^
		Mean ± SD	Median	Range	*p*-Value^1^	Mean ± SD	Median	Range	*p*-Value^2^	
WOMAC	E	45.9 ± 13.7 ^a^	42.0	19.0–65.0	<0.01	39.7 ± 12.8 ^a^	45.0	12.0–58.0	<0.01	<0.01
	MT.	46.3 ± 19.0 ^b^	44.0	2.0–81.0		40.1 ± 21.7 ^b^	33.0	1.0–84.0		0.01
	C	19.3 ± 17.6 ^a,b^	13.0	2.0–78.0		18.4 ± 18.6 ^a,b^	12.0	1.0–75.0		0.11 *

*p*-value^1^—the comparison of intergroup WOMAC pre-treatment (Kruskal-Wallis test or Mann-Whitney U test); *p*-value^2^—the comparison of intergroup WOMAC post-treatment (Kruskal-Wallis test or Mann-Whitney U test); *p*-value^3^—the comparison of intragroup WOMAC pre- and post-treatment (dependent t-student test or * Wilcoxon signed ranked test); ^a,b^—posthoc analysis.

**Table 4:** Results of VAS scales in E, MT, and C groups of participants.

Variable	Group	Before				After				*p*-Value^3^
		Mean ± SD	Median	Range	*p*-Value^1^	Mean ± SD	Median	Range	*p*-Value^2^	
VAS left	E	5.2 ± 2.7 ^a^	6.0	0–10.0	0.99	3.4 ± 2.1 ^a^	3.0	0–9.0	0.72	<0.01 *
	MT.	5.2 ± 2.4 ^b^	6.0	0–8.0		3.2 ± 2.7 ^b^	3.0	0–8.0		<0.01 *
VAS right	E	5.0 ± 2.7 ^a^	5.0	0–10.0	0.89	3.4 ± 2.2 ^a^	3.0	0–9.0	0.56	<0.01 *
	MT.	4.9 ± 2.7 ^b^	5.0	0–9.0		3.0 ± 2.6	2.0	0–8.0		<0.01

*p*-value^1^—the comparison of intergroup VAS pre-treatment (Kruskal-Wallis test or Mann–Whitney U test); *p*-value^2^—the comparison of intergroup VAS post-treatment (Kruskal-Wallis test or Mann-Whitney U test); *p*-value^3^—the comparison of intragroup VAS pre- and post-treatment (dependent t-student test or * Wilcoxon signed ranked test); ^a,b^—posthoc analysis.
